# New Perspectives of S-Adenosylmethionine (SAMe) Applications to Attenuate Fatty Acid-Induced Steatosis and Oxidative Stress in Hepatic and Endothelial Cells

**DOI:** 10.3390/molecules25184237

**Published:** 2020-09-15

**Authors:** Laura Vergani, Francesca Baldini, Mohamad Khalil, Adriana Voci, Pietro Putignano, Niccolò Miraglia

**Affiliations:** 1 Department of Earth, Environment and Life Science, University of Genoa, 16132 Genova, Italy; vocia@unige.it; 2 Department of Experimemtal Medicine, University of Genoa, 16132 Genova, Italy; baldinifrancesca92@gmail.com; 3 School of Pharmacy, University of Camerino, 62032 Camerino, Italy; mak_37_47@hotmail.com; 4 SP Diabetic Outpatient Clinic, ASST Monza, 20900 Monza, Italy; pietro.putignano@gmail.com; 5 Clinical & Pre-clinical Development, Gnosis by Lesaffre S.p.A, 20832 Desio, Italy; n.miraglia@gnosis.lesaffre.com

**Keywords:** S-adenosylmethionine (SAMe), non-alcoholic fatty liver disease, atherosclerosis, oxidative stress, steatosis, endothelium dysfunction

## Abstract

S-adenosylmethionine (SAMe) is an endogenous methyl donor derived from ATP and methionine that has pleiotropic functions. Most SAMe is synthetized and consumed in the liver, where it acts as the main methylating agent and in protection against the free radical toxicity. Previous studies have shown that the administration of SAMe as a supernutrient exerted many beneficial effects in various tissues, mainly in the liver. In the present study, we aimed to clarify the direct effects of SAMe on fatty acid-induced steatosis and oxidative stress in hepatic and endothelial cells. Hepatoma FaO cells and endothelial HECV cells exposed to a mixture of oleate/palmitate are reliable models for hepatic steatosis and endothelium dysfunction, respectively. Our findings indicate that SAMe was able to significantly ameliorate lipid accumulation and oxidative stress in hepatic cells, mainly through promoting mitochondrial fatty acid entry for β-oxidation and external triglyceride release. SAMe also reverted both lipid accumulation and oxidant production (i.e., ROS and NO) in endothelial cells. In conclusion, these outcomes suggest promising beneficial applications of SAMe as a nutraceutical for metabolic disorders occurring in fatty liver and endothelium dysfunction.

## 1. Introduction

S-adenosylmethionine (SAMe) is a pleiotropic endogenous metabolite acting as a co-substrate in transmethylation, transsulfuration, and aminopropylation reactions [1]. SAMe is mainly synthesized in the liver, and then distributed throughout the body [2], where it regulates many biochemical pathways, including the biosynthesis of hormones [3,4]. SAMe is the most important methyl donor in mammalian cells, where it transfers a methyl group to acceptor molecules, such as DNA, proteins, and lipids, thus modifying their structure and function [5]. Many reports have shown that SAMe treatment causes the hypermethylation of DNA, which is typically associated with the silencing of gene expression [6]. For this reason, SAMe has been proposed for use in cancer therapy to reduce tumor development, growth, and metastasis. Moreover, SAMe protects against oxidative stress as it is a precursor for cysteine, which is one of the amino acids of glutathione (GSH), and the major physiological defense against reactive oxygen species (ROS) [7].

Because of its pleiotropic functions, SAMe has been involved in many pathological conditions, especially liver disorders [5,8]. In patients with alcoholic hepatitis, reduced hepatic SAMe levels were described as being associated within decreased GSH levels [9], and SAMe administration was shown to normalize the GSH levels in the liver [10]. In general, SAMe treatment seems to protect against hepatic acute injury and fibrosis [11].

Non-alcoholic fatty liver disease (NAFLD) is the most common liver disease associated with obesity [12,13] and is correlated with type 2 diabetes and the cardiovascular risk [14]. Hepatic steatosis is the hallmark of NAFLD [15]. Although the hepatic storage of lipids is beneficial, excess hepatocyte enlargement may cause cell dysfunction [16]. Therefore, benign hepatic steatosis (NAFL) can progress to non-alcoholic steatohepatitis (NASH)—the inflammatory and necrotic form of liver steatosis at risk of developing fibrosis—until cirrhosis and hepatocellular carcinoma [15].

High levels of circulating fatty acids (FAs) are mediators of steatosis. In fact, excess FAs enter hepatocytes, where they can be esterified to triglycerides (TGs) and stored inside lipid droplets (LDs) as protection against their toxicity. Alternatively, FAs are metabolized in mitochondria and peroxisomes with the consequent production of reactive oxygen species (ROS). The oxidative stress resulting from stimulated fat catabolism promotes the progression of NAFLD to NASH [17,18]. LDs consist of a hydrophobic lipid core surrounded by a phospholipid monolayer and LD-associated proteins, which regulate lipid metabolism and traffic [19,20]. Among them, the adipose differentiation-related protein (ADRP) is crucial for the formation and structural maintenance of LDs and is a marker for the extent of lipid accumulation, as its overexpression stimulates lipogenesis and inhibits lipolysis [21].

NAFLD and endothelium dysfunction associated with atherosclerosis are comorbid conditions, particularly in individuals with metabolic syndrome [22]. A recent meta-analysis reported a significant association between NAFLD and endothelial dysfunction [23]. The biological mechanisms might involve insulin resistance, lipid dysmetabolism, and chronic inflammation, which are triggered by excess levels of fatty acids [24]. The endothelium is the first rate-limiting step in the utilization of long-chain FAs as fuels, and has many regulatory functions. Typically, endothelial cells respond to environmental signals through releasing various factors, including nitric oxide (NO) and ROS [25]. In the liver, the endothelial cells of sinusoids act in fibrosis development by sustaining the wound healing response and inflammation [26,27]. The wound healing process depends on endothelial cell migration, which is mediated by the intercellular adhesion molecule-1 (ICAM-1) on the plasma membrane [28].

Given SAMe’s excellent safety property, it is important to further clarify the mechanisms and efficacy of SAMe in NAFLD. In this study, we used rat hepatoma FaO cells exposed to a mixture of oleate/palmitate, which represent a reliable in vitro model for hepatic steatosis [29,30,31,32]. As FAs seem to have direct effects on oxidative stress of the vascular endothelium [33], we also used human endothelial HECV cells exposed to FAs that could be compared to in vivo atherosclerosis, which is typically observed in metabolic syndrome [34]. The results showed that SAMe ameliorated lipid accumulation in hepatic cells and reduced the lipid-dependent oxidative imbalance in both hepatic and endothelial cells, thus showing potential applications as therapeutic agents.

## 2. Materials and Methods

### 2.1. Chemicals

Unless otherwise indicated, the reagents employed were supplied by Sigma-Aldrich Corp. (Milan, Italy).

### 2.2. SAMe Preparation

**Figure 1:**
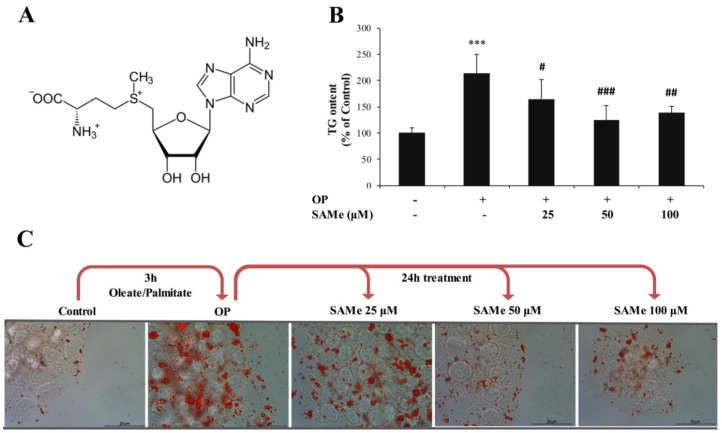
Effects of S-adenosylmethionine (SAMe) on lipid accumulation in FaO cells. FaO cells treated with 0.75 mM oleate/palmitate for 3 h (OP) were incubated with increasing concentrations of SAMe (25, 50, and 100 µM) for 24 h; the experimental control (Ctrl) consisted in FaO cells grown in the absence of OP and SAMe. (**A**) Chemical structure of SAMe; (**B**) intracellular TG content quantified by a spectrophotometric assay and expressed as the percentage of TG relative to the control; the TG content was normalized for the protein content determined by the Bradford assay. (**C**) Optical microphotographes of FaO cells stained with Oil-RedO (ORO) to label the cytosolic lipid droplets (LDs) (magnification 20x; Bar: 10 μm). Statistical significance between groups was assessed by ANOVA, followed by Tukey’s test. Symbols: C vs. all treatments: *** *p* ≤ 0.001; OP vs. all treatments: # *p* ≤ 0.05; ## *p* ≤ 0.01; and ### *p* ≤ 0.001.

S-adenosylmethionine (SAMe) (Figure 1A) was provided by Gnosis by Lesaffre (Desio, MB, Italy), as a salt of phytic acid. SAMe phytate was furnished as a dried powder. SAMe phytate is a novel salt of SAMe produced by Gnosis by Lesaffre (cat. N. 0-S34), with a batch-to-batch controlled profile on the basis of a defined specification sheet. The product is characterized by a SAMe phytate content ≥95.0% expressed on a dry basis and a total impurities content of ≤3.5% (expressed as the area percent), and both the titer and impurity profile are in agreement with the most common commercial salt—SAMe tosylate. The microbiology purity and heavy metal content of SAMe phytate preparation are in compliance with USP and Ph. Eur.

### 2.3. Cell Culture and Treatments

The FaO rat hepatoma cell line was supplied by the European Collection of Authenticated Cell Cultures (ECACC, Salisbury, Wiltshire, UK). FaO cells maintain many hepatocyte-specific markers [35]. FaO cells were grown at 37 °C with 5% CO_2_ in Coon’s modified Ham’s F12 medium supplemented with 10% fetal calf serum (FCS). HECV endothelial cells were supplied by Cell Bank and Culture (GMP-IST- Genoa, Italy). HECV cells were isolated from a human umbilical vein; they were grown at 37 °C in Dulbecco’s modified Eagle’s medium High Glucose (D-MEM) with 10% FCS. For treatments, all cells were grown until confluence (about 80%), and then incubated overnight in starvation medium (medium without serum, but with 0.25% bovine serum albumin (BSA)). To mimic in vitro the effect of a high-fat diet, cells were treated for 3 h with a mixture of two long-chain fatty acids—oleate and palmitate (2:1 molar ratio)—at a final concentration of 0.75 mM. Then, the lipid-loaded cells (OP) were incubated for 24 h in the absence or presence of SAMe (25, 50, and 100 µM). SAMe was prepared from the dilution of a 100 mM stock solution in 0.1 M HCl.

### 2.4. Protein Quantification

The protein content was determined by the bicinchoninic acid (BCA) method using BSA as a standard [36]. A Varian Cary50 spectrophotometer (Agilent, Milan, Italy) was employed.

### 2.5. Quantification of Triglycerides

Cells were scraped and centrifuged (14,000× *g* for 3 min). After cell lysis, lipids were extracted in chloroform/methanol (2:1) and chloroform was evaporated [29]. In each extract, the TG content was determined by spectrophotometric analysis using the Triglycerides Liquid Kit (Sentinel diagnostics, Milan, Italy). Values were normalized for the protein content determined by the BCA method. Data are expressed as the percent of TG content relative to controls.

### 2.6. ROS Production and Lipid Peroxidation Determination

To quantify in situ the production of H_2_O_2_ and other ROS, we employed the cell-permeant probe 2′-7′ dichlorofluorescin diacetate (DCF-DA), which is oxidized to 2′-7′dichlorofluorescein (DCF) in the cytosol [37]. DCF-DA was prepared from a stock solution (10 mM in DMSO) and stored at –20 °C in the dark. For the staining, cells were scraped, spun down (600× *g* for 10 min at 4 °C), and loaded with 10 µM DCF-DA in PBS for 30 min in the dark. After centrifugation, cells were suspended in PBS and the fluorescence was measured fluorometrically (λex = 495 nm; λem =5 25 nm) using an LS50B fluorimeter (Perkin Elmer, USA) at 25 °C with a water-thermostated cuvette holder.

Lipid peroxidation was determined spectrophotometrically through the thiobarbituric acid reactive substances (TBARS) assay using the reaction between malondialdehyde (MDA; 1,1,3,3-tetramethoxypropane) and thiobarbituric acid (TBA) [38]. Cell suspension was incubated for 45 min at 95 °C with a double volume of TBA solution (0.375% TBA, 15% trichloroacetic acid, 0.25 N HCl). Then, 1 vol. of N-butanol was added, the organic phase was collected, and the absorbance at 532 nm was recorded spectrophotometrically at 25 °C. For each sample, the MDA level was expressed as pmol MDA/mL/mg protein.

### 2.7. Oil-Red O Staining

The Oil-RedO (ORO) dye was used to stain the neutral lipids [39]. Cells were fixed in 4% paraformaldehyde, washed, and stained for 20 min with 0.3% ORO from a 0.5% stock solution in isopropanol. After washing, cells were examined by a Leica DMRB light microscope equipped with a DFC420C Leica CCD camera (Leica, Wetzlar, Germany).

### 2.8. Measurement of the Levels of Nitrites/Nitrates

Nitric oxide production was evaluated spectrophotometrically by quantifying the amount of nitrites and nitrates (NOx) in the samples through the Griess reaction [40]. The nitrite accumulation in the culture medium (μmol NaNO2/mg sample protein) was calculated against a standard curve of sodium nitrite (NaNO_2_). The absorbance at 540 nm was recorded at 25 °C with a Varian Cary 50 spectrophotometer. Data are the means ± S.D. of at least four independent experiments.

### 2.9. Real-Time qPCR

RNA isolated from the cells with Trizol reagent was retrotranscribed into cDNA. Then, the target mRNA was quantified by quantitative real-time PCR (qPCR) in quadruplicate using 1× IQTMSybrGreen SuperMix and the Chromo4TM System apparatus (Biorad, Milan, Italy) [29]. The comparative Cq method was employed to estimate the relative quantity of target mRNA, using glyceraldehyde 3-phosphate dehydrogenase (Gapdh) as the housekeeping gene. The mRNA expression was indicated as the fold induction with respect to controls [41]. Primer pair sequences are reported in Tables S1 and S2.

### 2.10. Wound Healing Assay

The Wound Healing assay allows the rate of cell migration to be assessed in vitro [28]. HECV cells were seeded on Petri dishes and incubated until confluence. Then, the cell monolayer was scraped with a pipet tip to create a ‘‘scratch’’, producing two crossing straight lines. Two images of the cross were acquired for each dish at 4× magnification. Then, the medium was replaced with fresh medium containing the SAMe at different concentrations (25, 50, and 100 µM), or with medium w/o SAMe (control). Sets of images were acquired at 0, 6, 24, and 48 h. To determine the rate of cell migration, the images were analysed with ImageJ free software (http://imagej.nih.gov/ij/). The size of the closed area as a function of time was expressed as the percentage compared with the value at time 0. Data are means ± S.D. of at least four independent experiments.

### 2.11. Statistical Analysis

Typically, data are expressed as means ± S.D. of at least four independent experiments in triplicate. Statistical analysis was performed using ANOVA with Tukey’s post-test (GraphPad Software, Inc., San Diego, CA, USA).

## 3. Results

### 3.1. Effects of SAMe on Lipid Accumulation in Hepatic Cells

The exposition of cultured cells to high concentrations of FAs mimic in vitro what occurs in vivo in different tissues during high-fat feeding and/or obesity. In this study, FaO cells were overloaded with lipids by exposure to an oleate/palmitate mixture (0.75 mM) for 3 h. Subsequently, cells were incubated for 24 h with the addition of different concentrations of SAMe (25, 50, and 100 µM). The cytotoxicity of SAMe at the concentrations under analysis was excluded by conducting a preliminary MTT assay (Figure S1 of Supplementary data). Moreover, we assessed the baseline effect of SAMe on lipid accumulation by exposing control FaO cells to SAMe and did not observe any significant effect (Figure S2 of Supplementary data).

**Figure 2:**
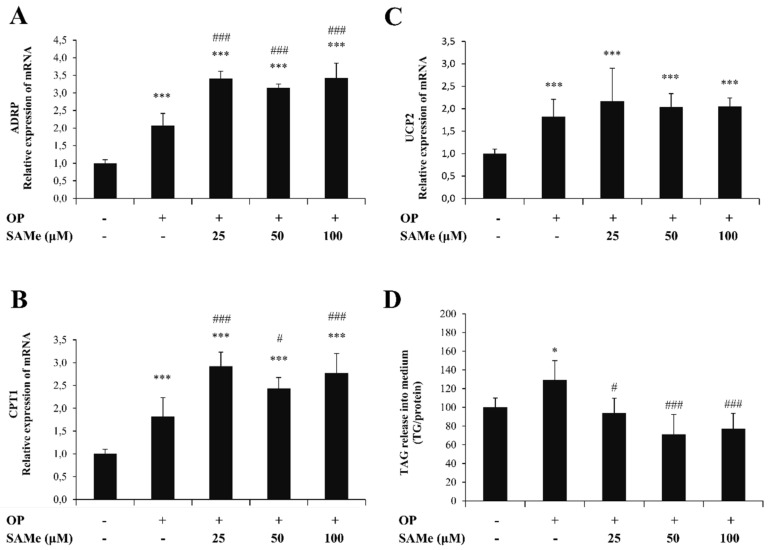
Effects of SAMe on lipid accumulation in FaO cells. We assessed the mRNA expression of (**A**) adipose differentiation-related protein (ADRP), (**B**) carnitine palmitoyltransferase 1 (CPT-1), and (**C**) uncoupling protein 2 (UCP-2) by quantitative PCR (qPCR); glyceraldehyde 3-phosphate dehydrogenase (GAPDH) was used as the internal control and data are expressed as the fold induction with respect to controls. (**D**) Extracellular TG content quantified in the medium by a spectrophotometric assay and expressed as the percentage of TG relative to the control. Statistical significance between groups was assessed by ANOVA, followed by Tukey’s test. Symbols: C vs. all treatments: * *p* ≤ 0.05 and *** *p* ≤ 0.001; OP vs. all treatments: # *p* ≤ 0.05 and ### *p* ≤ 0.001.

The intracellular TG content was quantified in control (C) and steatotic cells incubated in the absence (OP) or presence of SAMe. In lipid-loaded FaO cells (Figure 1B), we observed a significant increase in the TG content with respect to the control (+113%; *p* ≤ 0.001). This increase was significantly reverted upon incubation with SAMe 25, 50, and 100 µM (–49%, –89%, and –75%, respectively, compared to steatotic cells; *p* ≤ 0.05, *p* ≤ 0.001, and *p* ≤ 0.01, respectively). When the cytosolic LDs were visualized by ORO staining (Figure 1C), we observed that the number and size of LDs increased markedly in lipid-loaded cells (OP) compared to the control and decreased upon incubation with SAMe. ADRP is the main LD-associated protein in the liver cells which regulates the TG traffic from/to LDs. The ADRP mRNA level (Figure 2A) was significantly up-regulated in steatotic FaO cells (2.06-fold induction vs. control; *p* ≤ 0.001), and was further up-regulated after exposure to SAMe (about 3-fold induction vs. control; *p* ≤ 0.001 for all SAMe concentrations).

The extent of lipid accumulation in the liver depends on the balance between lipolytic and lipogenic pathways. We evaluated if and how the lipid-lowering action of SAMe was sustained by the stimulation of oxidative and/or secretory pathways of lipids. Lipolytic pathways lead to the final oxidation of FAs, mainly in mitochondria, where the mitochondrial protein CPT1 is a shuttle for FAs acting upstream of the mitochondrial FA oxidation. CPT1 mRNA expression was up-regulated in steatoic FaO cells (1.83-fold induction vs. control; *p* ≤ 0.001) and was further up-regulated upon exposure to SAMe (about 2.9-fold induction vs. control; *p* ≤ 0.001 for all SAMe concentrations) (Figure 2B). In mitochondria, UCP2 is an uncoupling protein that separates oxidative phosphorylation from ATP synthesis, resulting in energy dissipation as heat. Additionally, UCP2 expression was significantly up-regulated upon lipid-loading (1.82-fold induction vs. control; *p* ≤ 0.001) (Figure 2C), but SAMe at all concentrations did not significantly alter the UCP2 expression.

As an attempt to reduce TG accumulation, steatotic FaO cells stimulated the release of TGs into the medium (+29% with respect to control; *p* ≤ 0.05). Exposure to SAMe led to a significant reduction in TG release compared to steatotic cells (–35%, –58%, and –52%, for SAMe 25, 50, and 100 µM; *p* ≤ 0.05, *p* ≤ 0.001, and *p* ≤ 0.001, respectively) (Figure 2D).

### 3.2. Effects of SAMe on Oxidative Stress in Hepatic Cells

**Figure 3:**
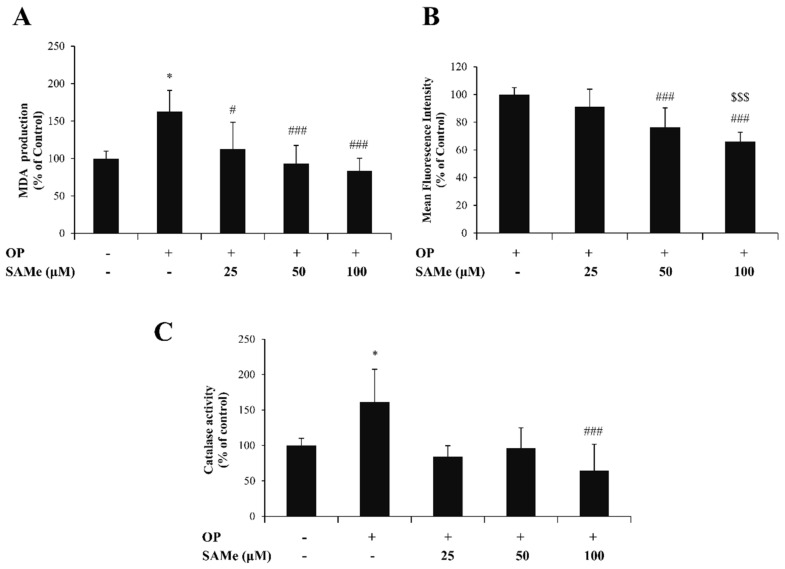
Effects of SAMe on oxidative stress in FaO cells. FaO cells treated with 0.75 mM oleate/palmitate for 3 h (OP) were incubated with increasing concentrations of SAMe (25, 50, and 100 µM) for 24 h; the experimental control consisted of FaO cells grown in the absence of OP and SAMe. We assessed (**A**) the intracellular level of malondialdehyde (MDA) (pmol MDA/mL × mg of sample protein) quantified by the thiobarbituric acid reactive substances (TBARS) assay; data are expressed as percentage values with respect to controls and normalized for total proteins. (**B**) The ROS levels were quantified by a fluorimetric assay of 2′-7′dichlorofluorescein (DCF)-stained cells; data are expressed as the percent mean fluorescence intensity (MFI) relative to steatotic cells and normalized for total proteins. (**C**) Catalase specific activity (micromoles of decomposed H_2_O_2_ per min/mg of sample protein) evaluated by a spectrophotometric assay, where data are expressed as percentage values with respect to controls and normalized for total proteins. Statistical significance between groups was assessed by ANOVA, followed by Tukey’s test. Symbols: C vs. all treatments: * *p* ≤ 0.05; OP vs. all treatments: # *p* ≤ 0.05 and ### *p* ≤ 0.001; OP+SAMe 25 µM vs. all treatments: $$$ *p* ≤ 0.001.

As a first indicator of the oxidative imbalance being potentially associated with lipid dysmetabolism, we assessed lipid peroxidation by the TBARS assay. As expected, the MDA level (Figure 3A) increased in steatotic FaO cells (+63% compared to control; *p* ≤ 0.05), and a dose-dependent decrease occurred upon exposure to SAMe compared to steatotic cells (–50%, –70%, and –79%, for doses of 25, 50, and 100 µM, respectively; *p* ≤ 0.05, *p* ≤ 0.01, and *p* ≤ 0.01, respectively).

Lipid peroxidation is typically caused by an excess of intracellular ROS, mainly hydrogen peroxide (H_2_O_2_), which we quantified by fluorimetric analysis (Figure 3B). Steatotic FaO cells (OP) incubated with SAMe showed a dose-dependent decrease in DCF florescence with respect to steatotic cells used as the control (–9%, –24%, and –34%, for doses of 25, 50, and 100 µM, respectively; *p* ≤ 0.001).

The changes in H_2_O_2_ levels were paralleled by changes in the enzymatic activity of catalase—the main enzyme catalyzing the H_2_O_2_ decomposition (Figure 3C). Catalase activity was stimulated in steatotic FaO cells (+61% compared to control) and was reduced upon the exposure of steatotic cells to SAMe (–77%, –65%, and –97%, for doses of 25, 50, and 100 µM, respectively; *p* ≤ 0.05 and *p* ≤ 0.01, respectively).

In control FaO cells, the MDA level, DCF signal, and catalase activity were not affected by SAMe (data not shown).

### 3.3. Effects of SAMe on Lipid Accumulation and Function in Endothelial Cells

HECV cells were overloaded with lipids by exposure to an oleate/palmitate mixture (0.75 mM) for 3 h. Cells were then incubated for 24 h with different doses of SAMe (25, 50, and 100 µM). The MTT assay was performed to exclude cytotoxic effects of the treatments on HECV cells (Figure S1 of Supplementary data). As preliminarily assessed, exposure to SAMe did not affect the TG level in control HECV cells (Figure S2 of Supplementary data).

**Figure 4:**
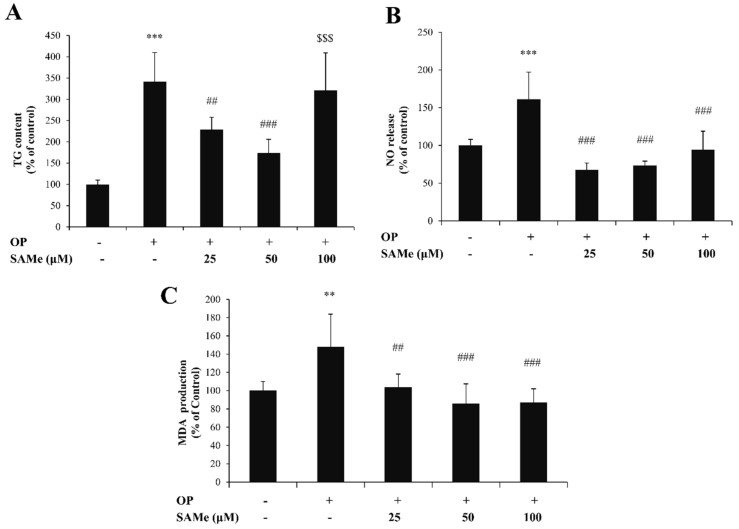
Effects of SAMe on lipid accumulation in HECV cells. HECV cells treated with 0.75 mM oleate/palmitate for 3 h (OP) were incubated with increasing concentrations of SAMe (25, 50, and 100 µM) for 24 h; the experimental control consisted of HECV cells grown in the absence of OP and SAMe. We assessed (**A**) the intracellular TG content expressed as a percentage of the TG content relative to the control; the TG content was normalized for total proteins determined with the Bradford assay; (**B**) NO release expressed as a percentage of NO release relative to the control, normalized for proteins; (**C**) the intracellular level of MDA (pmol MDA/mL × mg of sample protein) quantified by the TBARS assay; data are expressed as percentage values with respect to controls and normalized for total proteins. Data are means ± S.D. of four independent experiments. Statistical significance between groups was assessed by ANOVA, followed by Tukey’s test. Symbols: C vs. all treatments: ** *p* ≤ 0.01 and *** *p* ≤ 0.001; OP vs. all treatments: ## *p* ≤ 0.01 and ### *p* ≤ 0.001; OP+SAMe 50 µM vs. all treatments: $$$ *p* ≤ 0.001.

In lipid-loaded HECV cells (Figure 4A), we observed a significant increase in the TG content with respect to the control (+241%; *p* ≤ 0.001), and a significant decrease upon incubation with 25 µM (–33%; *p* ≤ 0.01) and 50 µM (–73%, *p* ≤ 0.001) SAMe compared to steatotic cells.

Nitric oxide (NO) is a major modulator of endothelial cell activity. In lipid-loaded HECV cells, we observed an increase in NO release with respect to the control (+61%; *p* ≤ 0.001), which was counteracted by SAMe at all concentrations of 25, 50, and 100 µM (–58%, –55%, and –41%, respectively, with respect to steatotic cells, *p* ≤ 0.001) (Figure 4B).

In endothelial cells, the lipid-loading also resulted in oxidative stress. The MDA level (Figure 4C) increased in lipid-loaded HECV cells (+48% compared to control; *p* ≤ 0.01), and decreased upon exposure to SAMe compared to steatotic cells (–32%, *p* ≤ 0.01; –42%, *p* ≤ 0.001; –41%, *p* ≤ 0.001; for doses of 25, 50, and 100 µM, respectively).

**Figure 5:**
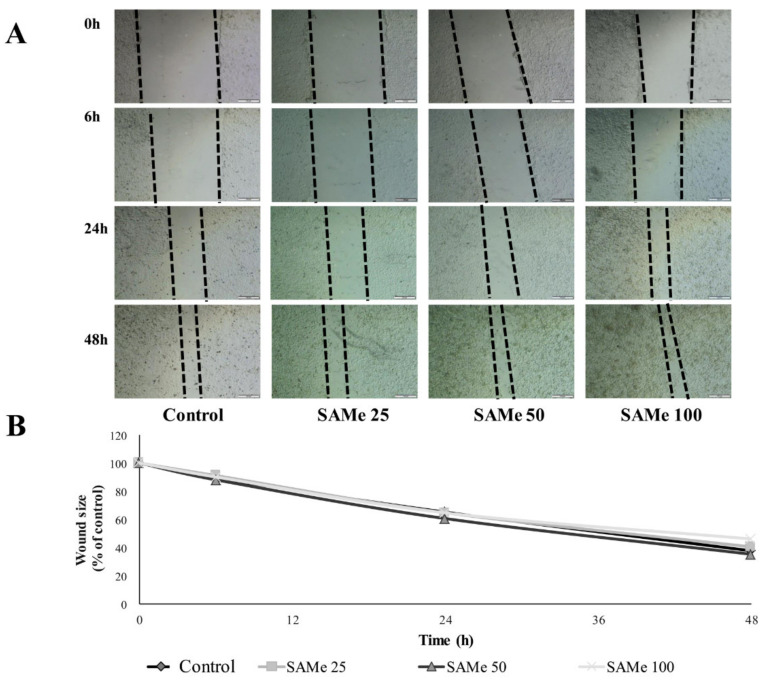
Effects of SAMe on HECV cell migration. The migration of HECV cells was examined using the Wound Healing assay. (**A**) Sets of images were acquired at 0, 6, 24 and 48 h. In order to determine the migration of cells in different conditions, the images were analysed using ImageJ free software (http://imagej.nih.gov/ij/). (**B**) Percentage of the closed area was measured and compared with the value obtained before treatment. An increase of the percentage of the closed area indicated the migration of cells. Data are means ± S.D. of four independent experiments. Statistical significance between groups was assessed by ANOVA, followed by Tukey’s test.

The SAMe effects on the migrating ability of HECV cells was evaluated using the Wound Healing assay (Figure 5). No significant differences in the cell migration rate were observed at all times after the scratch (for all doses of SAMe).

## 4. Discussion and Conclusions

SAMe—the main endogenous methyl donor—has many functions, including in the regulation of hepatocyte growth, differentiation, and death [8]. SAMe is also an important physiological defense against ROS over-production, mainly through the synthesis of GSH. In light of its pleiotropic activity, SAMe has been employed to improve liver abnormalities in several hepatic disorders [42]. The present in vitro study demonstrated a direct effect of SAMe in ameliorating fatty acid-induced steatosis and oxidative stress in hepatic and endothelial cells, and clarified some molecular mechanisms sustaining these effects.

Much evidence suggests that NAFLD and atherosclerosis share multiple cellular and molecular pathogenetic mechanisms [43,44]. In fact, the liver is both the target and source of systemic inflammatory factors [45]. Severe steatosis is associated with an increased atherosclerotic risk, depending on high circulating levels of inflammatory markers, such as C-reactive protein, interleukin-6 (IL-6), monocyte chemotactic protein 1, and Tumor Necrosis Factor α (TNF-α), as well as of procoagulant factors and oxidative stress mediators [46].

The cell models employed in the current study are hepatoma FaO cells and endothelial HECV cells exposed to an excess of exogenous fatty acids consisting of a mixture of oleate/palmitate, which are the most abundant FAs in both the human diet and body. The lipid-loaded cells are reliable in vitro models of fatty liver and endothelium dysfunction, respectively, which have been widely used in previous studies from our group [29,30,31,32].

In hepatocytes, an excess of exogenous fatty acids stimulated the cellular accumulation of TGs, resulting in hepatic steatosis visualized as an increase in the size and number of LDs. LDs are intracellular lipid reservoirs, but also provide building blocks for energy metabolism. In fact, FAs mobilized from LDs are directly utilized for mitochondrial and peroxisomal β-oxidation, and contact sites between LDs and mitochondria/peroxisomes allow the direct transfer of FAs. We found that steatosis in FaO hepatic cells was associated with the up-regulation of ADRP, CPT1, and UCP2 expression. ADRP is one of the most abundantly expressed LD-coating proteins promoting droplet formation [47]. CPT1 and UCP2 are two important mitochondrial proteins acting in FA oxidation and the energy balance: CPT1 transports long-chain fatty acids from the cytoplasm into the mitochondria for their β-oxidation, and UCP2 is an uncoupling protein that separates oxidative phosphorylation from ATP synthesis, resulting in energy dissipation as heat [48]. Therefore, the increased expression of CPT1 and UCP2 in steatotic hepatocytes appears as the attempt to limit excess fat accumulation by stimulating mitochondrial oxidation (CPT1), but avoiding excess ATP synthesis (UCP2). Moreover, in steatotic hepatocytes, we observed a higher generation of ROS which may be scavenged by antioxidant enzymes, but which might damage important macromolecules such as lipids, at least in part. Our data show that steatotic Fao cells stimulated catalase activity as protection against ROS over-production, but, despite this, the ROS excess, in part, may trigger the lipid peroxidation of membranes, which is a sign of oxidative stress.

On the other hand, in endothelial cells, the TG accumulation also resulted in a higher generation of ROS, as well as in stimulated catalase activity and increased lipid peroxidation. In addition to ROS generation, lipid accumulation in endothelial cells also stimulated the release of NO, which is a classical proinflammatory and vasodilatative signal [49]. We have to mention that high levels of NO may be detrimental for the vascular endothelium, for example, through its reaction with superoxide anions [50].

The exposure of steatotic hepatocytes to SAMe led to a decrease in TG accumulation through a reduction in the number and size of lipid droplets. Moreover, we also observed a further increase in ADRP expression when steatotic cells were incubated with SAMe. As previously described [51], ADRP overexpression could be a beneficial mechanism which may improve the insulin sensitivity. On the other hand, the reduced TG accumulation promoted by SAMe reflected on a reduced TG release into the extracellular medium. The lipid-lowering effect of SAMe seems to depend, at least partially, on the stimulation of mitochondrial oxidation, as suggested by the further up-regulation of CPT1 in steatotic cells exposed to SAMe at all concentrations. In contrast, UCP2 did not significantly change its expression upon SAMe incubation. Taken together, these results indicate that SAMe acts directly on steatotic hepatocytes by counteracting lipid accumulation and reducing the LD size and number, and this effect seems to depend on the acceleration of mitochondrial β-oxidation of long-chain FAs mediated by CPT1 activation.

We also observed that, in endothelial cells, exposure to SAMe led to a decrease in TG accumulation and in the oxidative imbalance being associated with FA accumulation. As a response to fat-depending dysfunction, endothelial cells stimulated the release of NO and the production of ROS, thus triggering a proinflammatory cascade. Our results showed that ROS production, as well as the stimulation of catalase activity, lipid peroxidation, and NO release, were counteracted by SAMe at all concentrations.

For some molecular parameters, we did not observe a dose-dependent effect of SAMe in the range of concentrations under analyses. However, the lack of a clear dose–response relationship has often been reported in vitro when the range of doses is rather narrow, as in the present study and in other papers of our group [52,53]. We wish to emphasize that, in the present study, we focused on SAMe concentrations that may have appreciable beneficial effects without any toxicity because of our interest in the nutraceutical potential of this molecule.

We can conclude that in both hepatic and endothelial cells, SAMe reduces fat accumulation and attenuates oxidative stress, protecting the cells from fatty acid-induced oxidative stress. Therefore, SAMe may be an efficacious lipid-lowering and antioxidant agent for steatotic hepatocytes and endotheliocytes.

In the liver, the antisteatotic effect of SAMe seems to be sustained by stimulating the fat catabolic pathways at a mitochondrial level, but without an increase in the oxidative stress. The direct protective effects of SAMe in vitro fit well with some previous studies conducted mostly in patients with alcoholic liver disease, cholestasis of pregnancy, and cirrhosis that showed significant improvements in liver test abnormalities during therapy with SAMe [11].

In the endothelium, the ability of SAMe in promoting endothelial cell migration may be important in attenuating or improving the endothelium dysfunction in pathological conditions. Indeed, it seems that the initiating event in the atherogenic process is some form of injury to the intimal endothelial lining, induced by noxious substances (e.g., oxidized cholesterol, hyperglycemia, etc.) or altered hemodynamic forces (e.g., blood flow disturbances due to hypertension). In particular, focal endothelial desquamation is envisioned as an inciting stimulus for platelet adhesion and the localized release of platelet-derived growth factors, which would then elicit the migration, proliferation, and phenotypic modulation of medial smooth muscle cells, thus generating a plaque.

In conclusion, our novel findings show the beneficial direct effect of SAMe as an antisteatotic and protective agent on two in vitro models of fatty liver and endothelium dysfunction, respectively, suggesting potential for this endogenous molecule as a nutraceutical for ameliorating these chronic pathological conditions.

## Supplementary Materials

The following are available online. Supplementary data include: Figure S1: Cell viability assay (MTT) of HECV and FaO cells after exposure to SAMe at the concentrations under analysis (0, 25, 50, and 100 µM) for 24 h was performed to exclude any cytotoxic effect. Figure S2: Intracellular triglyceride (TG) content of naïve FaO and HECV cells after exposure to SAMe at the highest concentration (100 µM) for 24 h was assessed to monitor the baseline effect of SAMe.
