# Supplementary material for: New Perspectives of S-Adenosylmethionine (SAMe) Applications to Attenuate Fatty Acid-Induced Steatosis and Oxidative Stress in Hepatic and Endothelial Cells
Source: Molecules. 2020 Sep 15;25(18):4237. doi: 10.3390/molecules25184237 (PMC7570632; doi:10.3390/molecules25184237)
Supplement: Supplementary file 1 [file molecules-25-04237-s001.pdf]

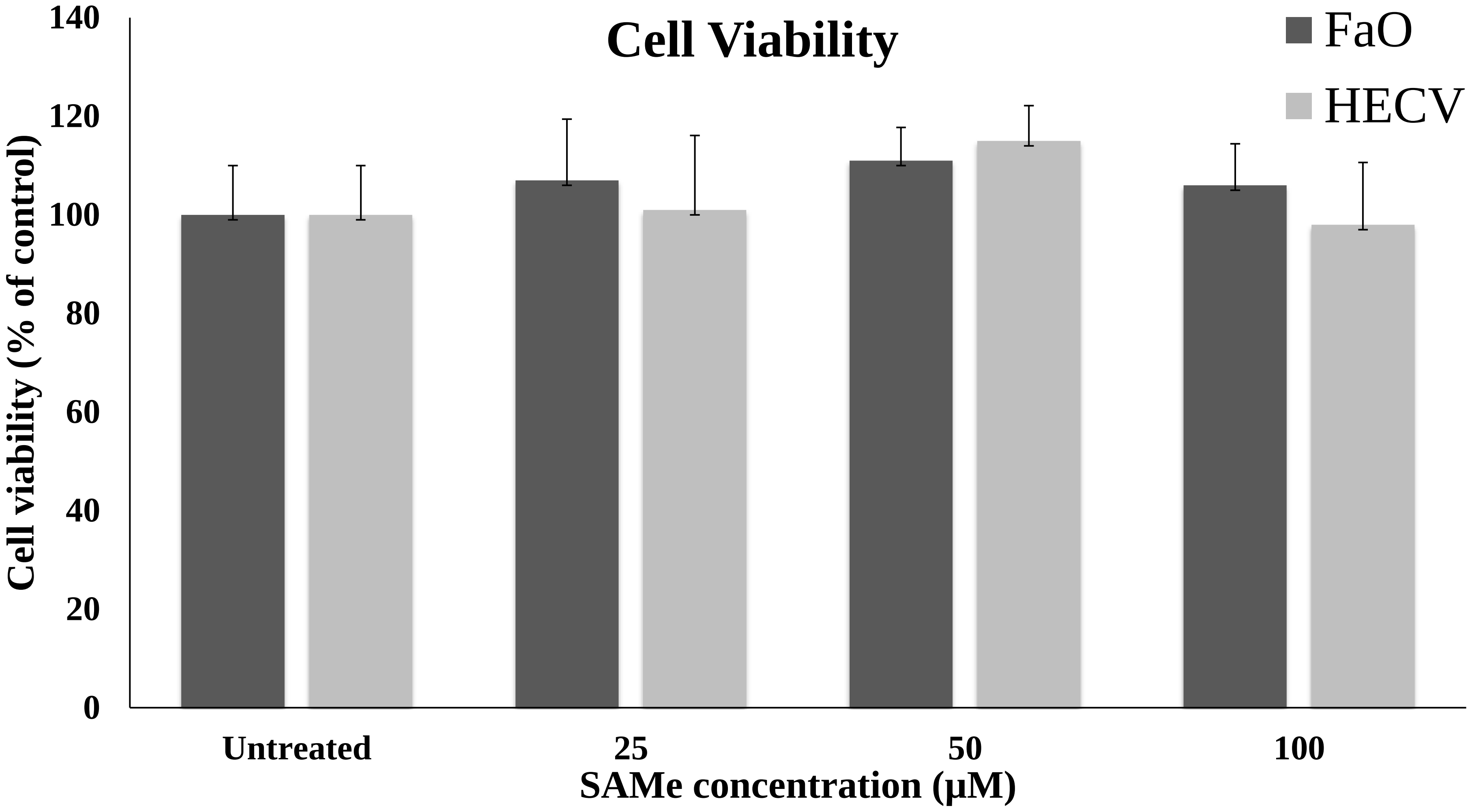

Figure 1S: Effect of SAMe on HECV and FaO cell viability: Cell were incubated with increasing concentrations of SAMe (25, 50 and 100 µM) for 24 h; the experimental control (C) consisted in HECV cells grown in the absence of SAMe. We assessed the cell viability by MTT assay. Data are expressed as percentage values with respect to controls. Data are means±S.D. of four independent experiments.

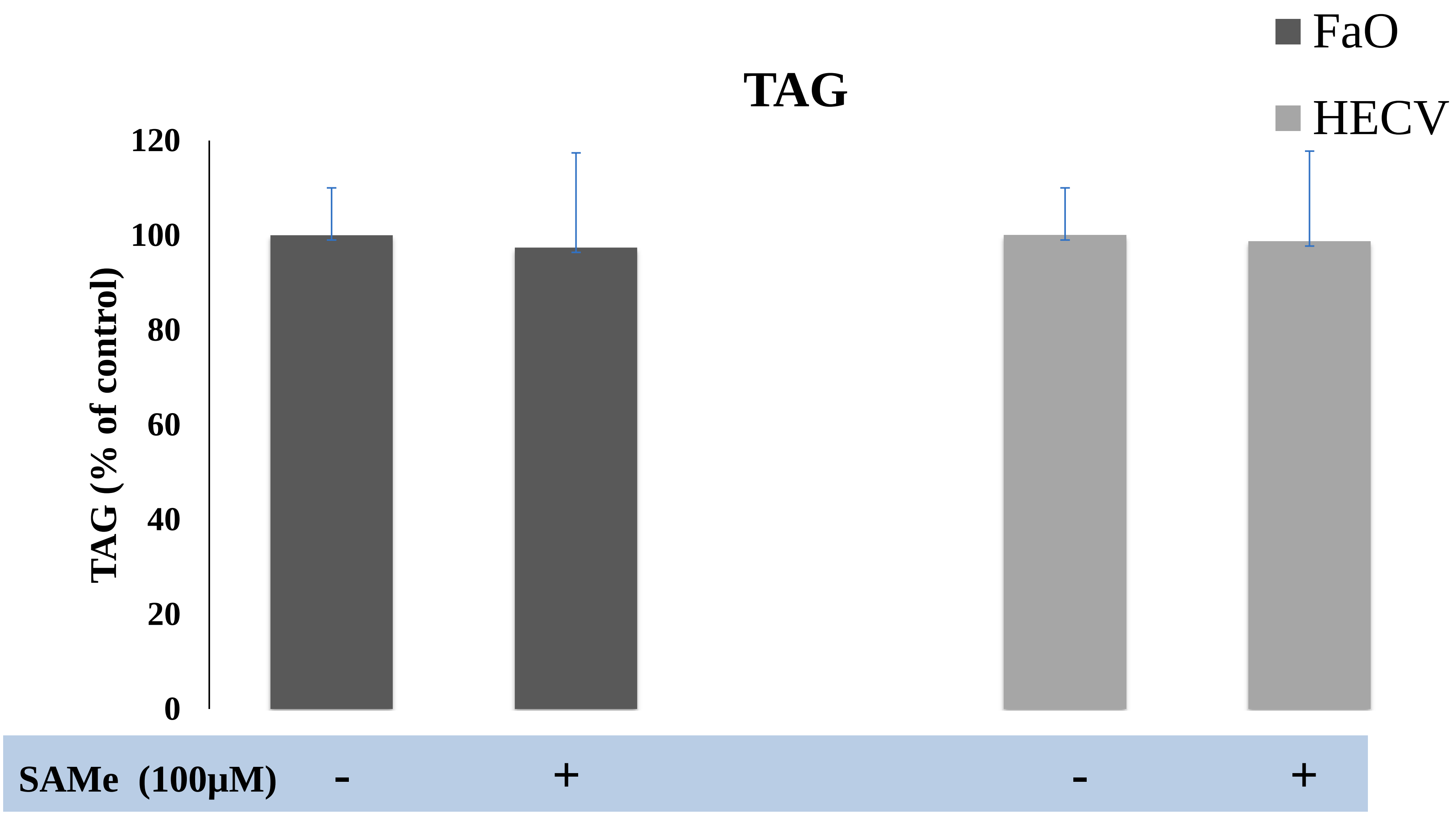

Figure 2S: Effect of SAMe on triglyceride accumulation. FaO and HECV cells were treated with SAMe at 100 µM for 24 h; the experimental control (C) consisted in cells grown in the absence of SAMe. We assessed the intracellular TG content expressed as percentage of TG content relative to control; TG content was normalized for total proteins determined with Bradford assay. Data are means±S.D. of three independent experiments.
